# Unmet needs of the Iranian mothers of the children with cancer and the identification of the related factors: A descriptive-correlational study

**DOI:** 10.3389/fpsyg.2022.964424

**Published:** 2022-09-23

**Authors:** Leila Khanali Mojen, Maryam Rassouli, Hadis Ashrafizadeh, Ensieh Fathollah Zadeh, Nasrin Dadashi, Tahereh Alsadat Khoubbin Khoshnazar, Parand Pourazarhagh, Tahereh Nasrabadi

**Affiliations:** ^1^Pediatric Congenital Hematologic Disorders Research Center, Research Institute for Children’s Health, Shahid Beheshti University of Medical Sciences, Tehran, Iran; ^2^Cancer Research Center, Shahid Beheshti University of Medical Sciences, Tehran, Iran; ^3^Student Research Committee, Faculty of Nursing, Dezful University of Medical Sciences, Dezful, Iran; ^4^College of Nursing and Health Sciences, Flinders University, Adelaide, SA, Australia; ^5^Department of Medical Surgical Nursing, School of Nursing and Midwifery, Shahid Beheshti University of Medical Sciences, Tehran, Iran; ^6^School of Nursing and Midwifery, Iran University of Medical Sciences, Tehran, Iran; ^7^Mofid Hospital, Tehran Medical Sciences Branch, Islamic Azad University, Tehran, Iran; ^8^Department of Nursing, Faculty of Nursing and Midwifery, Tehran Medical Sciences, Islamic Azad University, Tehran, Iran

**Keywords:** cancer, care, child/children, parents/family, caregivers, unmet needs

## Abstract

**Introduction:**

Parents’ caring responsibilities lead to imbalances in roles, functions, and emotions, confronting them with new needs that, if left unaddressed, have adverse consequences for the lives of all family members. Therefore, this study aimed to identify the significant unmet needs of the mothers of the children with cancer.

**Materials and methods:**

This descriptive cross-sectional study was conducted in Tehran in 2019–2020 on 215 mothers of the children with cancer visiting the hospitals affiliated with medical universities. The study population was selected through convenience sampling method and according to the inclusion criteria. The FIN questionnaire consisting of two subscales, FIN-Import and FIN-Fulfillment, with 40 items, was used to collect data.

**Results:**

The mean age of the mothers participating in the study was 34.77 ± 7.91 years, and the mean age of the children with cancer was 9.77 ± 14.37 years. The mean scores of FIN-Import and FIN-Fulfillment were 92.88 ± 7.97 and 70.82 ± 17.89, respectively. The phrase “To know the facts concerning my child’s prognosis” with 23%, and the phrase “To be told about the people who could help with problems” with 22.8% were the most common unmet needs reported by the parents.

**Conclusion:**

The present study indicated that caring for a child with cancer had confronted mothers with new needs to be met; however, not all of these needs had been satisfied. Awareness about the unmet needs of these mothers and planning to manage those needs can lay the groundwork to improve their health.

## Introduction

Cancer, as a chronic, debilitating, and common disease, is the second leading cause of death due to non-communicable diseases in the age group under 20 years (Global Burden of Disease Study, 2017; World Health Organisation, 2018). In Iran, the prevalence of cancer in children has significantly increased in recent years. According to the available evidence, Iran has the highest incidence rate of childhood cancers in Asia (Ferlay et al., 2018). Although advances in medical science and improved treatment methods have increased the survival of affected children (Ahmadi Pishkuhi et al., 2018), they have posed challenges to the long-term care of these survivors. The physical, psychological, and emotional consequences of cancer and its treatment can significantly alter a child’s ability and influence their experiences of returning to life and pre-diagnosis routines (Li et al., 2013).

Cancer Diagnosis in a child is one of the most distressing and challenging life experiences for parents, which causes psychological complications and severe emotional stress (Pirbodaghi et al., 2016) as well as numerous changes in all their roles (Mendenhall and Katherine, 2018). This imposes more duties and responsibilities than parental responsibilities, including assisting the sick child in daily activities, providing emotional support, assisting him/her in the management of the illness or treatment, and supporting him/her (Fergus et al., 2015; Zimmermann, 2015). These duties are often performed without prior training, sufficient knowledge, or receiving support (Fergus et al., 2015; Regan et al., 2015). The complex process of caring for children with cancer can impact the physical, mental, social, economic, and marital health of parents, and the persistence of such condition can diminish their life quality (Salmani et al., 2014; Kim and Yi, 2015). Consequently, family members are required to reorganize their roles, interactive patterns, and relationships inside and outside the family and endeavor to adapt to the new situation (Panganiban-Corales and Medina, 2011).

As a result of various effects of cancer on the family, parents experience diverse needs, many of which may be overlooked, and these unmet needs might have immediate consequences for the sick child and the family (Miller et al., 2013; Litt and McCormick, 2015). Awareness of family needs and efforts in order to satisfy them reduce the parental care burden (Mohd Nordin et al., 2019). Since mothers are considered the child’s primary caregiver, the quality of care for the sick child is improved through meeting the mother’s needs. Therefore, it is indispensable to identify parents’ needs in order to meet them (Hallström et al., 2002; Seyedamini, 2011).

Family caregivers of cancer patients have countless unmet needs (Cui et al., 2014; Sklenarova et al., 2015). For instance, the parents of the children with leukemia need the information to learn how to provide care for their child in terms of physical, mental, and lifestyle needs (Motlagh et al., 2019) as well as disease management and treatment methods (Maree et al., 2016), hopefulness, and receiving sufficient information (Bužgová et al., 2016). The medical information on cancer, treatment methods, and physical, mental, and educational health (Borjalilu et al., 2017) was among the parents’ most significant needs according to the literature.

The concerns of family members, severe stress, and unmet needs may impair not only effective communication and collaboration with health professionals during hospitalization, but also the ability of families to provide psychological and spiritual support to patients in the later stages of the illness (Deeken et al., 2003). To provide effective and quality care, it is necessary to identify the problems and the unmet needs of the family members during the child’s hospitalization. This is the only way in which professional caregivers can provide adequate intervention and purposive palliative care (Hwang et al., 2003).

On the other hand, it seems that some variables or characteristics can impact parents’ needs and their dissatisfaction. In other words, to help meet parents’ needs, it is necessary to identify and adjust the variables that hinder the satisfaction of needs. Furthermore, identifying the mothers with these characteristics causes caregivers to consider them as a specific group in need of particular attention and pay greater attention to meet their needs. According to the study’s results, there is a correlation between the unmet needs of the parents of the children with cancer and educational level, economic status, the number of children, children’s treatment status, life quality, and family employment status (Arabiat and Altamimi, 2013; Bužgová et al., 2016; Motlagh et al., 2019).

Although in recent years, many studies have been conducted in the world on the unique needs of the parents of the children with cancer (Kerr et al., 2007; Rietveld et al., 2019), very little research in Iran has focused on perceiving and evaluating the needs of parents. However, the results of these studies also provide insights into the stressors in parents and the cultural perspectives on caring for a child with cancer in Iran (Yin and Twinn, 2004; Wong and Chan, 2006; Ji et al., 2018). The results of a study in Iran, which examined the information needs of the parents of the children with cancer in the form of a qualitative study, show that the greatest concern in relation to this group of children is identifying their health needs regarding cancer (Borjalilu et al., 2017). The studies conducted on the caring ability of the mothers of the children with cancer also shows that one of the factors weakening the caring ability of families is their low awareness of caring for these children (Nemati et al., 2018; Khademi et al., 2019), which reminds the need to pay attention to the unmet needs of the mothers of the children with cancer. In addition, some studies have emphasized age, sex, socioeconomic status, education, and the length of care as factors influencing the responses of parents, as the primary source of care (Munsell and Cohen, 2010). However, given that parents’ experiences of caring for a child with cancer is influenced by cultural and social conditions, it is logical that the individual conditions of the caregiver, other demographic characteristics of the parents, and the clinical condition of the child be considered as the predictors of mothers’ needs, too (Munsell and Cohen, 2010; Stajduhar et al., 2011).

Identifying the needs of mothers and caregivers along with the needs of the patient is an important care priority because both the family and the patient should be considered as a unit of care. Therefore, one of the care goals is to identify the needs and their effective predictors that have led to tension, high levels of stress, and frustration in families, which can peace and adjustment for families can ultimately be achieved through meeting these needs (Nachshen, 2005; Garrouste-Orgeas et al., 2010).

By altering the role of family members following a child’s developing cancer and caring for him/her, unknown challenges are created in family roles and relationships; therefore, raising experienced needs. Considering that the results of qualitative and quantitative studies conducted on the health of Iranian mothers of the children with cancer have indicated the low life quality of these mothers (Jadidi et al., 2013; Borjalilu et al., 2017; Rezaei et al., 2018), they seem to have unmet needs or require serious support. Identifying these needs might reduce their concerns and care burden and increase their life quality, thus enabling them to provide comprehensive care for a child with cancer. Therefore, this study was conducted to determine the unmet needs of Iranian mothers of the children with cancer and identify the factors associated with these needs.

### Conceptual framework

Conceptualizing this study is based on the research of Kristjanson et al. (1995), which measures the care needs of the families with cancer patients. Considering the importance of the needs of this group of caregivers, they have designed the FIN instrument in two dimensions: FIN-Importance of Care Needs and FIN-Fulfillment of Care Needs (Kristjanson et al., 1995). In Khademi et al. (2022) study, this tool has been psychometrically evaluated for the mothers of the children with cancer, which had acceptable validity and reliability (Khademi et al., 2022). Given that the FIN instrument has been psychometrically evaluated to reflect the characteristics of the social and cultural system governing the mothers of the children with cancer in Iran, and according to the psychometric properties of the FIN instrument, efforts were done to use this instrument to assess the needs of the mothers of the children with cancer.

## Materials and methods

### Study design and environment

This is a descriptive-correlational study conducted in Iran from October 2019 to April 2020. The study environment consisted of five children’s hospitals affiliated with medical universities in Tehran (the capital city of Iran) and equipped. These hospitals are the referral centers for the children with cancer from all over the country and represent a community of the Iranian mothers of the children with cancer.

### Participants

The study population included all the mothers of the children with cancer visiting the above mentioned centers, who were selected through convenience sampling method and based on the inclusion criteria. The required sample size was considered at least 200 individuals using the sample size formula ($n=\frac{Z^{2}\,p\,q}{d^{2}}$), taking into account the test power of 0.8, type 1 error of 0.05, and 5% sample attrition. The inclusion criteria consisted of mothers’ awareness of the definitive diagnosis of childhood cancer, fluency in Farsi, six months having passed since the diagnosis of the child’s disease in order to pass the shock and the reactions due to the diagnosis of the disease, no history of psychiatric disorders in mothers based on self-report, as well as their sick child’s being in the age range of 1–17.

This group of children in Iran are hospitalized in pediatric wards and under the supervision of a pediatric oncologist. The age group under one year was not included in the study due to different hospitalization and care conditions.

Due to the different needs of children with cancer in the later stages of life and consequently the different needs of mothers, because of possible differences in the needs and the conditions of meeting them, the mothers who also had another child with cancer or a child in the later stages life were not included in the study.

### Measurements

The data collection instrument consisted of two sections: the demographic and clinical status questionnaire and the FIN (Family Inventory Need).

The demographic and clinical status questionnaire consists of questions regarding the mother’s age, educational level, job, marital status, and income level, as well as the child’s age, gender, and stage of disease, the number of children, the number of hospitalization times, the type of cancer, the treatment method, and the time of diagnosis.

FIN was first developed by Kristjanson et al. (1995) in with 40 items in two versions. The FIN-Importance version has been designed to measure the importance of the family care needs of cancer patients, and the FIN-Fulfillment Needs Care version, to measure the extent to which family care needs are met (Kristjanson et al., 1995). The 40-item instrument was first translated, and its psychometric properties were investigated by Khademi et al. (2022) in on Iranian mothers with cancer. Cronbach’s alpha for FIN-Import and FIN- Fulfillment was α = 0.90 and α = 0.94, respectively. The ICC for the original version and between two tests was obtained ICC = 0.91 (Khademi et al., 2022).

FIN-Import determines the importance of each need, which is rated using a 5-point Likert scale from one (Not important at all) to 5 (Very important). The lowest and the highest scores are 20 and 100, respectively; the highest score indicates greater importance. FIN-Fulfillment measures the satisfaction rate of needs that received a score higher than one in the first section on a 3-point Likert scale [Met (5), partly met (3), and Unmet (1)]. The lowest and the highest scores are 20 and 100, respectively; the higher score indicates that the family needs are met (Kristjanson et al., 1995).

To collect the data, the questionnaires were distributed among the mothers who met the inclusion criteria and whose children were hospitalized and under treatment. The approximate time to complete each questionnaire was estimated to be about 20 minutes. Sampling was performed during both morning and evening shifts for two months. At the time of questionnaire completion by the mother, the researcher was present to answer her possible questions.

### Statistical analysis

In this study, the data were analyzed using SPSS-V24 software. Kolmogorov-Smirnov statistical test was used to test the normality of the data. The tables of frequency distribution, central tendency (mean and median), and the indices of dispersion (variance and standard deviation) were used to describe the data. Spearman and Pearson’s correlation tests were done to determine the correlations between FIN subscales and quantitative variables. Finally, linear regression was applied to determine the relationships between the variables.

### Ethical considerations

This study has been approved by the ethics committee of Shahid Beheshti University of Medical Sciences (IR.SBMU.PHARMACY.REC. 1398.049). The purpose of the study was explained to the mothers. They were informed about the aim of the study, and filled the questionnaires with full consent. They were also reassured about the confidentiality of the information and the possibility of withdrawing from the study at any stage. All the procedures in the study of the human participants took place in accordance with the ethical standards of the National Research Committee and the 1964 Declaration of Helsinki.

## Results

Based on the study findings, out of 215 mothers participating in the study, 210 filled out the questionnaire, and the response rate was calculated to be 97%. The mean age of the mothers participating in the study was 34.55 ± 7.15 years, with the lowest and the highest ages of 17 and 57, respectively (CI = [33.57, 35.53]). The time of diagnosis was 17.91 ± 19.44 months. Other demographic and clinical features are listed in Table 1.

**TABLE 1:** Demographic and clinical variables of mothers with children with cancer.

Variables		Number	Percent
Education	High School	96	45.7
	Diploma	68	32.4
	Academic	43	20.5
Job	Unemployed	5	2.4
	Free (Azad)	21	10
	Employee	18	8.6
	housewife	161	76.7
	Other	5	2.4
Marital status	Married	180	85.5
	Divorce	22	10.5
	Widow	7	3.3
Income level	Enough	18	8.6
	medium	119	56.7
	Insufficient	72	34.3
Child’s gender	girl	107	51
	boy	103	49
Type of cancer	leukemia	108	51.4
	Glioma	6	2.9
	Neuroblastoma	4	1.9
	Lymphoma	22	10.5
	Osteosarcoma	20	9.5
	Nephroblastoma	3	1.4
	Others	35	17.2
Number of hospitalizations	Once	33	15.7
	2–4	42	20
	More than of forth	134	63.8
Stage of disease	Stage 1	63	30
	Stage 2	47	22.4
	Stage 3	47	22.4
	Stage 4	2	1
Treatment method	Chemotherapy	139	66.2
	Radiotherapy	2	1
	Surgery	10	4.8
	Chemotherapy + Radiotherapy	20	9.5
	Chemotherapy + Surgery	18	8.6
	Other	19	9

The mean score of the ‘important needs’ subscale (FIN-Import) was 92.88 ± 7.97, with minimum and maximum ranges of 67 and 100, respectively (CI = [91.85, 94.03]). Based on the results of this study, 77.5–99.5% of the mothers reported the needs as very important (Table 2).

**TABLE 2:** The importance of the needs of mothers with children with cancer.

FIN-Import	Not important		Somewhat important		Average importance		Very important		Extremely important	
					
	Number	Percent	Number	Percent	Number	Percent	Number	Percent	Number	Percent
Have my questions answered honestly	1	1.2	0	0	1	1.2	24	27.9	60	69.8
Know specific facts concerning the patient’s prognosis	0	0	0	0	3	3.5	16	18.6	67	77.9
Feel that the health professionals care about the patient	0	0	0	0	4	4.7	17	19.8	65	75.6
Be informed of changes in the patient’s condition	0	0	0	0	2	2.3	17	19.8	67	77.9
Know exactly what is being done for the patient	0	0	0	0	4	4.7	21	24.4	61	70.9
Know what treatment the patient is receiving	0	0	0	0	3	3.5	21	24.4	62	72.1
Have explanations given in terms that are understandable	0	0	0	0	2	2.3	22	25.6	62	72.1
Be told about treatment plans while they are being made	0	0	2	2.3	2	2.3	20	23.3	62	72.1
Feel there is hope	1	1.2	0	0	6	7	6	7	73	84.9
Be assured the best possible care is being given to the patient	0	0	0	0	2	2.3	12	14	72	83.7
Know what symptoms the treatment or disease can cause	0	0	0	0	5	5.8	16	18.6	65	75.6
Know when to expect symptoms to occur	0	0	0	0	7	8.1	13	15.1	66	76.7
Know the probable outcome of the patient’s illness	0	0	0	0	3	3.5	18	20.9	65	75.6
Know why things are being done for the patient	0	0	3	3.5	2	2.3	14	16.3	67	77.9
Know the names of health professionals involved in the patient’s care	7	8.1	5	5.8	5	5.8	22	25.6	47	54.7
Have information about what to do for the patient at home	0	0	0	0	0	0	25	29.1	61	70.9
Feel accepted by the health professionals	4	4.7	5	5.8	7	8.1	25	29.1	45	52.3
Help with the patient’s care	0	0	1	1.2	2	2.3	16	18.6	67	77.9
Have someone be concerned with my health	8	9.3	8	9.3	15	17.4	21	24.4	34	39.5
Be told about people who could help with problems	8	9.3	6	7	10	11.6	21	24.4	41	47.7

The mean score of the ‘unmet needs’ subscale (FIN- Fulfillment) was 70.82 ± 17.89, with minimum and maximum ranges of 20 and 100, respectively (CI = [68.12, 73.13]). Findings showed that 30–50% of the mothers reported their needs as unmet or partly met. The most common unmet needs reported by mothers were “The need to know the specific facts concerning the patient’s prognosis” (23.3%), and, in the second rank, “To be told about the people who could help with problems” (22.8%). Their highest satisfied need was “Help with the patient’s care” (68.1%) (Table 3).

**TABLE 3:** Unmet needs of mothers with children with cancer.

FIN-Fulment	Unmet		Partly met		Met	
			
	Number	Percent	Number	Percent	Number	Percent
Have my questions answered honestly	23	11	110	52.4	71	33.8
Know specific facts concerning the patient’s prognosis	49	23.3	114	54.3	47	22.4
Feel that the health professionals care about the patient	26	12.4	106	50.5	74	35.2
Be informed of changes in the patient’s condition	23	11	117	55.7	67	31.9
Know exactly what is being done for the patient	28	13.3	93	44.3	86	41.0
Know what treatment the patient is receiving	30	14.3	67	31.9	110	52.4
Have explanations given in terms that are understandable	41	19.5	102	48.6	64	30.5
Be told about treatment plans while they are being made	32	15.2	78	37.1	97	46.2
Feel there is hope	34	16.2	94	44.8	79	37.6
Be assured the best possible care is being given to the patient	18	8.6	92	43.8	97	46.2
Know what symptoms the treatment or disease can cause	26	12.4	94	44.8	85	40.5
Know when to expect symptoms to occur	27	12.9	106	50.5	73	34.8
Know the probable outcome of the patient’s illness	34	16.2	106	50.5	66	31.4
Know why things are being done for the patient	33	15.7	102	48.6	71	33.8
Know the names of health professionals involved in the patient’s care	13	6.2	75	35.7	122	58.1
Have information about what to do for the patient at home	35	16.7	88	41.9	84	40
Feel accepted by the health professionals	22	10.5	65	31	119	56.7
Help with the patient’s care	13	6.2	54	25.7	143	68.1
Have someone be concerned with my health	19	9	109	51.9	79	37.6
Be told about people who could help with problems	48	22.8	106	50.3	56	26.7

Pearson correlation test showed that there is no statistically significant correlation between the mean FIN-Import score with the mother’s age (*p* = 0.599, *r* = –0.037), the child’s age (*p* = 0.629, *r* = 0.034), and the time of diagnosis (*p* = 0.829, *r* = 0.016). There was not a statistically significant correlation between the mean FIN-Fulment score and the mother’s age (*p* = 0.052, *r* = –0.137), the child’ age (*p* = 0.211, *r* = 0.087), and the time of diagnosis, either (*p* = 0.754, *r* = –0.022).

The results of univariate regression to examine the relationship between the demographic and the clinical variables with the mean scores of FIN-Fulment and FIN-Import are shown in Table 4. According to the regression coefficients, the mean FIN-Import score has a significant relationship with marital status (widow and separated), the type of cancer (lymphoma, osteosarcoma, etc.), the third stage of the disease and chemotherapy treatment along with surgery. Considering that the significant values mentioned for these variables are less than 0.05, it can be said that these variables are good predictors for the dependent variable or the average FIN-Import score. The coefficient of determination (R2) was equal to 0.117, which indicates that 11.7% of the changes in the mean FIN-Import were explained by demographic and clinical variables (F = 7.44, Sig = 0.000, R = 0.368, R square = 0.135).

**TABLE 4:** Result of univariate regression between demographic and professional characteristics and total score of FIN-Import and FIN-Fulment.

Outcomes	Total score of FIN-Import					Total score FIN-Fulment				
Parameter	Beta	SE	95% CI for Beta	*t*	*P*	Beta	SE	95% CI for Beta	*T*	*P*
**Marital status**										
Married	Ref	–	–	–	–	Ref	–	–	–	–
Divorced	3.57	1.72	[0.20, 6.94]	4.31	0.038	3.52	4.10	[–4.52, 11.57]	0.73	0.391
Widow	6.14	2.93	[0.39, 11.89]	4.38	0.036	–6.14	6.86	[–19.59, 7.30]	0.80	0.371
**Educational status**										
High school	Ref	–	–	–	–	Ref	–	–	–	–
Diploma	–1.08	1.26	[–3.55, 1.38]	0.74	0.389	–1.94	2.81	[–7.46, 3.57]	0.47	0.490
Academic	–0.431	1.45	[–3.29, 2.42]	0.08	0.768	3.21	3.28	[–3.23, 9.65]	0.95	0.328
**Job**										
Unemployed	Ref	–	–	–	–	Ref	–	–	–	–
Freelance	0.38	3.87	[–7.12, 7.97]	0.01	0.922	–17.54	8.77	[–34.73, –0.35]	3.99	0.462
Employee	–0.50	3.93	[–8.21, 7.21]	0.01	0.899	–16.62	8.91	[–34.08, 0.844]	3.47	0.047
Housewife	–3.98	3.53	[–10.91, 2.94]	1.26	0.260	–15.92	8.00	[–31.62, –0.231]	3.95	0.062
Other	–2.40	4.92	[–12.05, 7.25]	0.23	0.626	–8.20	11.14	[–30.05, 13.65]	0.54	0.046
**Income Level**										
Enough	Ref	–	–	–	–	Ref	–	–	–	–
Medium	–1.55	2.01	[–5.50, 2.39]	0.59	0.440	–8.81	4.46	[–17.57, –0.05]	3.88	0.049
Insufficient	–1.50	2.09	[–5.61, 2.61]	0.51	0.474	–8.87	4.67	[–18.02, 0.27]	3.61	0.057
**Child’s gender**										
Girl	Ref	–	–	–	–	Ref	–	–	–	–
Boy	–0.47	1.10	[–2.63, 1.69]	0.18	0.669	6.50	2.44	[1.71, 11.62]	7.08	0.008
**Type of cancer**										
Leukemia	Ref	–	–	–	–	Ref	–	–	–	–
Glioma	3.59	3.11	[–2.50, 9.69]	1.33	0.248	–1.86	7.21	[–16.00, 12.27]	0.06	0.796
Neuroblastoma	1.42	3.77	[–5.97, 8.82]	0.14	0.706	–3.69	8.75	[–20.85, 13.45]	0.17	0.673
Lymphoma	6.79	1.73	[3.38, 10.19]	15.31	0.000	–10.06	4.02	[–17.95, –2.17]	6.24	0.012
Osteosarcoma	3.47	1.80	[–0.06, 7.01]	3.70	0.054	–4.49	4.18	[–12.71, 3.71]	1.15	0.283
Nephroblastoma	2.92	4.34	[–5.58, 11.43]	0.45	0.500	–9.03	10.06	[–28.75, 10.69]	0.80	0.369
Others	3.72	1.44	[0.89, 6.55]	6.67	0.010	–9.89	3.35	[–16.46, –3.31]	8.27	0.003
**Number of hospitalization**										
Once	Ref	–	–	–	–	Ref	–	–	–	–
2–4	1.55	1.80	[–1.98, 5.09]	0.73	0.390	8.51	4.11	[0.45, 16.58]	4.29	0.004
More than of forth	–2.68	1.51	[–5.64, 0.27]	3.15	0.076	9.86	3.45	[3.10, 16.63]	8.16	0.038
**Stage of disease**										
Stage 1	Ref	–	–	–	–	Ref	–	–	–	–
Stage 2	–1.60	1.53	[–4.61, 1.39]	1.10	0.294	–5.53	3.38	[–12.17, 1.11]	2.66	0.103
Stage 3	–4.69	1.53	[–7.69, –1.68]	9.37	0.002	3.80	3.41	[–2.87, 10.49]	1.24	0.264
Stage 4	–0.84	5.71	[–12.03, 10.35]	0.02	0.883	–6.19	12.59	[–30.87, 18.48]	0.24	0.623
**Treatment of method**										
Chemotherapy	Ref	–	–	–	–	Ref	–	–	–	–
Radiotherapy	4.48	5.55	[–6.39, 15.36]	0.65	0.419	–1.02	12.17	[–24.88, 22.82]	0.007	0.933
Surgery	4.78	2.55	[–0.21, 9.78]	3.51	0.061	–6.02	5.59	[–17.00, 4.94]	1.16	0.282
Chemotherapy + Radiotherapy	0.12	1.82	[–3.44, 3.70]	0.05	0.944	–15.64	4.00	[–23.50, 7.79]	15.25	0.000
Chemotherapy + Surgery	3.83	1.86	[0.18, 7.48]	4.23	0.040	–5.32	4.09	[–13.35, 2.69]	1.69	0.193
Other	2.59	1.95	[–1.22, 6.42]	1.76	0.183	–9.25	4.28	[–17.65, 0.85]	4.65	0.031

In addition, according to the regression coefficients, the mean FIN-Fulment score has a significant relationship with the mother’s occupation (employee, etc.), income level (average, insufficient), the patient’s gender (male), the type of cancer (lymphoma, osteosarcoma), the number of hospitalizations, chemotherapy along with radiotherapy, etc. Considering that these variables’ significance values are less than 0.05, it can be said that these variables are good predictors of FIN-Fulment. The coefficient of determination (R2) was equal to 0.175, which indicates that 17.5% of the changes in the mean FIN-Fulment were explained by demographic and clinical variables (F = 8.715, Sig = 0.000, R = 0.445, R square = 0.198).

## Discussion

This study was conducted to identify the unmet needs of the mothers of the children with cancer, and showed that despite the great importance of all these needs from most mothers’ perspectives, 30–50% of them were unmet or partly met. The need to know the truth about the child’s prognosis and support sources to help the mother when confronted with problems were the most common unmet needs expressed by the mothers. The partial or incomplete satisfaction of needs is a global concept or challenge. In different countries, based on access to various services and socio-economic variables, the priority of parents’ needs and their satisfaction rate vary (Lyu et al., 2019).

Parental care responsibilities lead to imbalances in roles, functions, and emotions, and expose them to unknown needs (Fergus et al., 2015; Zimmermann, 2015). Most parents’ care needs are related to information, emotional, psychosocial, practical, spiritual, and physical fields, respectively, which are satisfied through supportive and comfort care (Koohkan et al., 2019). In Iran, comfort care for children is a novel concept, and there are no organized services for these patients and their families (Mojen et al., 2018). Furthermore, most Iranian parents complain about the high care burden and constantly seek support and comfort services (Ahmadi et al., 2018). Therefore, the dissatisfaction of their needs is anticipated. In line with these results, previous studies conducted in other populations likewise pointed to the multiple needs of mothers of children with cancer, and the rate of need dis satisfaction was reported to be 22–56% (Friðriksdóttir et al., 2011; Arabiat and Altamimi, 2013; Aziza et al., 2019; Lyu et al., 2019).

In this study, the mothers’ most common unmet need was “To know specific facts concerning the patient’s prognosis.” In this regard, previous studies have shown that parents expected to know the facts about their child’s illness. Parents are confused when their child is diagnosed with cancer, and constantly search for information about the disease type, treatment, and outcome (Moridi et al., 2018; Ahmadnia et al., 2021).

Uncertainty over the diagnosis of cancer in a child is one of the challenges for parents. Uncertainty refers to the inability to determine the meaning of disease-related events or to predict the consequences of the disease, which leads to fear and anxiety in patients and caregivers. The complications and adverse effects of the disease make patients unconfident about their prognosis, and they, along with their caregivers, are constantly in an aura of fear and uncertainty about what might occur in the future (Lie et al., 2018). According to various studies in Iran, one of the experiences of the mothers of the children with cancer is expecting the child’s imminent death, which might be due to a lack of information about the child’s prognosis (Nikfarid et al., 2015; Mojen et al., 2018; Moridi et al., 2018).

Another unmet need of mothers was “To be told about the people who could help with problems.” In Iran, patients are left unaccompanied after discharge, and there is no integrated follow-up service for them. Access to health information sources and the ability to communicate with the treatment team is among the needs of the caregivers of a child with cancer (Hashemi et al., 2014; Koohkan et al., 2019). Due to time restrictions, physicians are generally unable to establish a satisfactory therapeutic relationship with the patient and the family, and cannot provide sufficient information about the child’s illness to parents (Rozveh et al., 2017). Meanwhile, the family considers the physician to be the most reliable source of information. If the family receives information from a source other than physicians, they will be dissatisfied and continue to complain about the lack of support provided for their child care and consider it as an unmet need (Friðriksdóttir et al., 2011). Available informational support resources are among the strategies to meet these needs. The results of several studies on the caregivers of cancer patients in Iran showed that most caregivers lack the necessary knowledge to provide adequate and safe care, adapt to their caregiving role, and reduce stress, which may be due to the lack of specific education and support system (Nikfarid et al., 2015; Khademi et al., 2019; Motlagh et al., 2019).

Despite policymakers’ emphasis, the provision of services at the community level is fundamental, and deficiencies such as the lack of a proper structure, the lack of systematized services as comfort care, the lack of family contribution, and legal, security, and financial issues are the most significant reasons for the dissatisfaction of parents’ needs (Hemati et al., 2016; Rassouli et al., 2017). In other words, satisfying the needs of the mothers of the children with cancer is possible through providing comfort care and taking it into account from various aspects.

The mothers of the children with cancer believed that the greatest satisfied need was to have the ability to help with the patient’s care. In line with the present study results, Arabiat et al. reported that the most common satisfied need of the mothers of the children with cancer was the need to participate in child care.

They and the care team were equally involved in care provision (Arabiat and Altamimi, 2013). The child’s independence in self-care compels mothers to take some supportive measures and participate in care provision (Mojen et al., 2018). In the Iranian culture, the family is of utmost value, and parents have a high sense of responsibility towards their children. In other words, their participation in child care is a symbol of family strength. Participation in child care requires the empowerment of mothers (Vasli and Salsali, 2014). Although one of the primary needs of mothers was the need for information to make them empowered, based on various studies, mothers acknowledged that they had gained invaluable experiences in caring for their children from their peer groups (Mojen et al., 2018; Moridi et al., 2018).

Since identifying the factors that can affect the important and unmet needs of the mothers of the children with cancer is considered an important step in addressing these needs, in the present study, some predictors of mothers’ needs have been investigated.

Examining the relationship between clinical variables and needs showed that there is a significant relationship between the number of hospitalizations, the stage of the disease and the type of pediatric cancer with the mean score of FIN (Importance, Fulfillment). In other words, if the number of hospitalizations increases and the child with cancer is in a more advanced stage, the mother will have more unmet needs, and the needs will be more important in these situations. Bužgová et al. (2016) showed that, as the child’s disease progresses, the likelihood of repeated hospitalization increases and the mother experiences difficult conditions (Bužgová et al., 2016). Obviously, in this situation, the child with cancer requires extensive care.

The results of several studies confirm this finding of the present study: in the advanced stages of the disease, cancer patients and their caregivers have more physical and mental needs, and significantly a lower quality of life (So et al., 2013, 2014; Edib et al., 2016) because as the disease progresses, longer and more complex treatments will be needed. Besides, unforeseen side effects lead to numerous physical and mental problems and alter the individual’s adaptability. Poor mental adaptability may increase physical disability or vice versa (Akechi et al., 2011). The presence and persistence of the disease forces families to have to change their areas of function. In addition, in some cases, the strategies they take to deal with and control the disease are ineffective or not applied properly. This reduces their caring ability over time compared to the onset of the disease. Iranian mothers, despite being under great pressure and the many responsibilities that they have, are less often involved in deciding on the type of treatment chosen for their children; most decisions are made by fathers. This imposes a double burden on the mother (Khademi et al., 2019). Moreover, the inability of the mother to spend time with the family, due to the many care needs of the sick child, is another reason behind the care burden that results from their many and complex child care responsibilities. Therefore, these mothers face many challenges and needs caring for their children at home.

Another result of this study was the existence of a significant relationship between the family’s income level and their unmet needs. Kim and Yi (2015) showed that the level of household income is related to the unmet needs of cancer patients’ caregivers, which affects their quality of life (Kim and Yi, 2015). In other studies, the unmet needs of patients and low-income caregivers were found to be greater (Houts et al., 1988; Liu, 2008). Another study showed that the higher the income level and the economic situation of families, the lower their information needs (Motlagh et al., 2019). However, in some studies, the income level has been found to have little to do with unmet needs (Liu, 2008; Hasegawa et al., 2016). This variation may be due to the heterogeneity of the studies conducted in different fields of culture, health systems, and economic levels, which may be related to unmet needs. This finding can be justified by the fact that most caregivers are forced to quit their jobs to provide full-time care to their sick children, and this will increase their financial burden, which will eventually lead to new challenges (Golant and Haskins, 2008). Cancer treatment and related financial pressures can be a powerful source of stress for patients and their families, especially for low-income patients (Rassouli and Sajjadi, 2016). This issue, itself, is one of the most important unmet needs of the parents of the children with cancer and is a reminder of the need for financial support provided by both public and private sectors as well as charities.

Another factor influencing the FIN-Important of the mothers was their marital status: the needs of divorced and widowed mothers were greater than those of married mothers. In a study conducted in Japan, a statistically significant relationship was reported between marital status and the unmet support needs of cancer patients (Okamura et al., 2021), while in the study by Khademi et al. and Loureiro et al. no statistically significant relationship was found (Loureiro et al., 2013; Khademi et al., 2019). Although few studies have pointed to the relationship between these two variables, it can be said losing a spouse or separating from him/her, as a very great stressor, can upset the emotional and mental balance of family members, which is more common in women due to their personality type and, sometimes, they have to take the responsibility of caring for their children by themselves.

If there is a sick child, this stress and tension will double and they will may feel helpless due to social and economic pressures (Haffariyan et al., 2007). Therefore, it is natural for the divorced and the widowed mothers of the children with cancer to experience more unmet needs due to lack of a support system and their emotional-mental imbalance.

### Limitation

Although the mothers in this study were selected from pediatric cancer hospitals in Tehran (the capital of Iran), which are the referral centers from all over the country, the samples may not represent the entire population of the mothers of the children with cancer throughout Iran. Moreover, the not including mothers in the first two stages after diagnosis and the end of life stage was another limitation of the present study.

### Implication of practice

Due to the importance of identifying the unmet needs of mothers, it is necessary to identify these needs at each visit and design a care program to meet them. If necessary, the mother must be referred to receive specialty services. The results of this study can help professionals to identify the unmet needs of the mothers of the children with cancer and to pave the way for the provision of family-centered nursing care to support parents, especially mothers, in the face of stress. The first step in empowering families is a needs assessment. By using appropriate educational interventions such as family therapy, training parents, and providing enough space for the dynamism of the family environment in interaction with the medical staff, their unmet needs can be satisfied. Considering that the dimension of awareness and knowledge in the mothers participating in the study was the most unmet need mentioned by them, it is recommended that the treatment team use strategies to meet mothers’ educational and care provision needs in order to prevent a decrease in their caring ability.

## Conclusion

The results showed that mothers, due to the nature of the child’s disease and their role in care provision, considered all the needs important, and the needs’ being unmet or partially met was among their reported challenges. The most common unmet need was associated with the information about the child’s disease. These findings highlight the necessity to provide support services as well as educational and family empowerment programs. It is recommended that planning for family-centered nursing care, with emphasis on recognizing family needs, be initiated for the parents of the children with cancer. Consequently, effective measures can be taken to enhance family empowerment. Furthermore, it is suggested that nursing care be provided based on the nursing process so that comprehensive investigation and recognition is performed, and parents’ knowledge deficit and unawareness is identified. Given the possible differences in parents’ needs in the early stages of the disease due to the lack of adaptability and stability, it is suggested that future research be conducted to identify these unmet needs in the early stages of the disease. Considering that there is no palliative care provision system in Iran and since continuous care is not provided from the beginning to the end of life, it is recommended that the unmet needs of the mothers with the children in the final stages of life be examined.

## Data availability statement

The original contributions presented in this study are included in the article/supplementary material, further inquiries can be directed to the corresponding author.

## Ethics statement

This study was approved by the Ethics Committee of Shahid Beheshti University of Medical Sciences (IR.SBMU.PHARMACY.REC. 1398.049). The purpose of the study was explained to the mothers. They were informed about the aim of the study, and filled the questionnaires with full consent. They were also reassured about the confidentiality of the information and the possibility of withdrawing from the study at any stage. All the procedures in the study of the human participants took place in accordance with the ethical standards of the National Research Committee and the 1964 Declaration of Helsinki. The patients/participants provided their written informed consent to participate in this study.

## Author contributions

LK, MR, HA, EF, ND, TAKK, TN, and PP involved in the study conception and design, in critical revisions for important intellectual content and administrative and technical support, and supervised the work. LK, MR, ND, and HA contributed to the data collection and analysis. LK, MR, and HA drafted the manuscript. All authors contributed to the article and approved the submitted version.
